# Mapping Online Discourse on Topical Steroid Withdrawal: Accuracy, Safety, and Supporting Evidence

**DOI:** 10.7759/cureus.96024

**Published:** 2025-11-03

**Authors:** Joyce J Zhu, Annabel Hou, Jillian Baader, Zoe Lipman, Kayla Fourzali, Sharon E Albers

**Affiliations:** 1 Department of Dermatology, University of South Florida Morsani College of Medicine, Tampa, USA

**Keywords:** content analysis, drug response, social media analysis, topical steroid misuse, topical steroid withdrawal

## Abstract

Introduction

Topical steroid withdrawal (TSW) refers to a collection of adverse effects related to the cessation of topical corticosteroid use, especially with prolonged use or higher potency formulations. While TSW lacks official recognition and standardized diagnostic criteria, the topic has gained significant attention online. This study analyzes social media discussions surrounding TSW to gain an improved understanding of patient perspectives and assesses the accuracy and safety of the online discourse.

Methods

TSW-related content was retrieved from social media platforms Instagram and TikTok. Educational content and content specifically created by physicians were rated in accuracy, safety, and agreement by dermatology residents. From Reddit, TSW-related posts in the Subreddit r/eczema were extracted and then analyzed with a sentiment analysis tool.

Results

The majority of online discourse consisted of users sharing their personal experiences. Educational content consistently fell below the midpoint for accuracy, safety, and agreement on Instagram and TikTok, whereas physician-generated content averaged 4.25 out of 5 in all three categories. Online users discussed non-conventional treatments, including cold atmospheric plasma therapy and no moisture therapy, that present with limited evidence in the management of TSW. Dermatologist-generated content discussed the potential use of dupilumab, which has been utilized in case studies for the treatment of TSW.

Conclusion

The presence of unregulated and potentially harmful treatment advice in current discourse can pose risks to patient well-being. Establishing standardized diagnostic criteria, prioritizing research into TSW management, and increasing dermatologist involvement online are crucial steps in improving patient care and safety in the context of TSW.

## Introduction

Topical steroid withdrawal (TSW) and topical steroid addiction (TSA), commonly used interchangeably, describe a constellation of adverse effects experienced by some patients following the discontinuation of topical corticosteroids (TCS). While no formally accepted definition exists, TSW is characterized by a developed physical dependency on corticosteroids and an exacerbation of symptoms upon cessation. TCS are frequently prescribed by dermatologists to manage a variety of inflammatory skin conditions and are a mainstay of treatment for atopic dermatitis, allergic contact dermatitis, psoriasis, and vitiligo [1]. Symptoms of TWS often overlap with those of the original treated disease, particularly atopic dermatitis or other eczematous eruptions, which can complicate the diagnosis. Symptoms of TWS include erythema, pruritus, burning, vesiculation, telangiectasia, and skin atrophy [2,3]. Patients may also experience symptoms relating to thermal dysregulation such as facial hot flashes [3]. More specific signs associated with TWS include the “red sleeve” sign described as an eczematous eruption that abruptly ends at the proximal dorsal and palmar hand, thickened skin with reduced elasticity on the face and genitals termed “elephant wrinkles,” and the “headlight” sign described as an eruption over the face sparing the mid-facial triangle created by the nose and upper lip [2,4]. These three signs were reported at rates of 40%, 56%, and 29%, respectively, in a study of 69 patients [4].

The duration of TCS use associated with the onset of TSW varies, ranging from two months to 40 years of mid- to high-potency formulation use [2]. The risk of developing TSW increases with prolonged use, application over larger surface areas, higher potency formulations, and treatment of thin-skinned areas such as the face and genitals [5]. It is hypothesized that children and women are especially susceptible [6]. Although the pathogenesis of TSW remains unconfirmed, there are various postulated mechanisms for its development. One such explanation is tachyphylaxis, which is the diminishing response and increased insensitivity to TCS when used extensively [2].

Other hypotheses include dysregulated cortisol production, dysregulated glucocorticoid action, rebound vasodilation, and rebound cytokines secondary to barrier disruption. Full spontaneous recovery from TSW after complete cessation of steroids can take weeks to years, as skin sensitivity gradually lessens and skin returns to its baseline status [7]. Reports have also highlighted the psychological effects of TWS, including insomnia, depression, anxiety, and suicidal thoughts [6].

The diagnosis of TWS remains a topic of debate within the dermatological community. One study recommends the following features to be used as essential diagnostic criteria for TSW: history of long-term and regular TCS use, itch, and erythema, supported by the presence of additional symptoms as discussed earlier [4]. TSW is not formally recognized within standardized coding systems like the International Classification of Diseases (ICD). Physicians' hesitancy to validate the condition may stem from the limited availability of high-quality research regarding TSW and the lack of diagnostic guidelines to distinguish TSW from exacerbation of the underlying inflammatory disorder being treated with TCS [4]. TSW is not recognized as a disease entity in traditional dermatologic texts and, if taught in residency, is in the context of an undesired outcome of inappropriate topical steroid use. In a 2024 survey of British Association of Dermatologists members, only 11.6% of respondents felt “very confident” addressing patient concerns about TSW, while 61.2% believed it reflected a typical eczema relapse [8]. Nonetheless, TSW has gained more traction in recent years, as evidenced by its recognition by the National Eczema Association and ongoing research by National Institute of Health researchers on establishing provisional diagnostic criteria for TSW [9].

Despite the dermatology community’s hesitancy to recognize TWS as a diagnosis, the popularity of TWS as a patient-recognized diagnosis has increased significantly in recent years. From 2016 to 2020, mentions of TSW on social media platforms increased by 274% [10]. Patients have recognized their symptoms in others with TWS in online communities, driving many to share their experiences along with their feelings of dismissal by and distrust of the dermatology community who does not fully recognize their condition. In the medical literature, one case report detailed the experience of a patient with self-diagnosed TSW after six months of unsuccessful treatment for a debilitating and worsening rash; she was met with skepticism upon discussing TSW with her healthcare providers [11]. While social media can serve as a valuable resource for patients to find community and support, it may facilitate misinformation that leads to increased patient harm. Lack of physician guidance for TWS leaves patients vulnerable to potentially harmful alternative therapies and "steroid phobia," where individuals may discontinue necessary treatments out of fear [12]. Such actions can exacerbate underlying skin conditions or contribute to the development of TSW symptoms. In a cohort study, all patients who presented to their dermatology healthcare providers with online research were pursuing non-conventional treatments [6]. These included no moisture regimens, diet changes, and treatment by overseas providers.

For optimal patient care, it is important for the dermatology community to better understand the patient-directed information and discourse about TWS in online communities and social networks. This study aims to characterize the nature of TSW-related online discussions across various social media platforms, identifying the predominant types of discourse, investigating the alternative therapies promoted online, and assessing the quality of information between physician and non-physician-generated content. By examining these interactions, this study seeks to help bridge the gap between patient experiences and clinical perspectives.

## Materials and methods

Posts regarding TSW on social media platforms Instagram and TikTok were retrieved on June 19, 2024. Relevant posts were identified on Instagram using the hashtag “#topicalsteroidwithdrawal” and on TikTok using the search “topical steroid withdrawal.” From each platform, the first 100 posts that met the inclusion criteria were obtained. The inclusion criteria were that TSW was the main subject of the post and was in the English language. Product advertisements and sponsorships were excluded.

The 100 obtained posts from each social media platform were reviewed and categorized into one of the three categories: educational, sharing personal experience, and artistic expression. Posts that provided recommendations or medical advice to viewers were classified as educational. Posts that described or showed personal experiences with TSW without offering recommendations were classified as personal experience. Finally, posts that used creative or visual media, such as illustrations related to TSW, were classified as artistic expression. Educational posts were rated separately by two dermatology residents. Each of these posts was rated on a scale of 1-5 on accuracy, safety, and agreement (the extent to which the resident agreed with the information in the post). The accuracy, safety, and agreement scores for each platform were averaged, yielding three total scores for each platform. Calculations were repeated for content specifically created by physicians.

Reddit social media posts from the Subreddit r/eczema were also extracted for analysis. Data were extracted on June 20, 2024, using Python (Python Software Foundation, Fredericksburg, VA, US), an open-source programming language widely used in software development and data science, and Python Reddit API Wrapper (PRAW), a Python package that facilitates easy access to Reddit’s interface. Text posts from the top 1,000 submissions on the r/eczema Subreddit were filtered for the keywords “TSW” or “topical steroid withdrawal” to identify and extract relevant posts. The corresponding comments for these posts were also extracted.

Sentiment analysis was performed on the Reddit posts and comments using Valence Aware Dictionary and sEntiment Reasoner (VADER), an open-source lexicon and rule-based sentiment analysis tool specifically attuned to social media content [13]. VADER returns an aggregate sentiment score between the values of -1 (indicating most negative) and +1 (indicating most positive) that represents the overall sentiment of the given text. Cutoff values were set as follows: less than or equal to -0.05 as negative, between -0.05 and 0.05 as neutral, and greater than or equal to +0.05 as positive. Of the extracted posts, those that received a sentiment score of less than or equal to -0.05, corresponding to negative sentiment, were reviewed and classified into categories describing the topics of the posts.

## Results

Of the 100 posts obtained from Instagram, 14 (14%) were educational, 82 (82%) were about the user’s personal experiences, and four (4%) were some form of artistic expression, such as drawing a portrait of themself with TSW. None of the posts were made by physicians. The average scores for the accuracy, safety, and agreement of the educational posts were 2.64, 2.93, and 2.68 on a scale of 1 to 5 rated by dermatology residents. All three scores were below 3, the midpoint of the scale.

**Figure 1:**
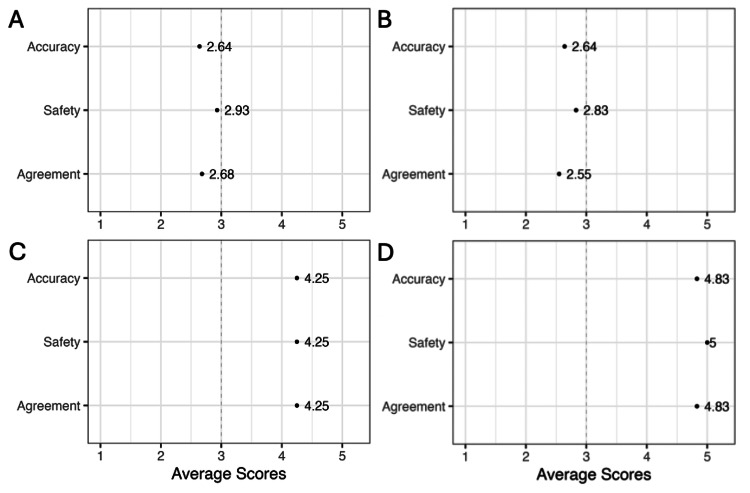
Average scores for accuracy, safety, and agreement of educational posts on Instagram and TikTok The average ratings were calculated for educational posts on (A) Instagram and (B) TikTok, as well as for TikTok posts (C) created specifically by physicians and (D) created specifically by dermatologists. Ratings were determined by two independent dermatology residents on a scale of 1-5 in three categories: accuracy, safety, and clinical agreement.

Of the 100 posts obtained from TikTok, 15 (15%) were educational, 84 (84%) were about the user’s personal experiences, and one (1%) fell under artistic expression. Four (4%) of the posts were made by physicians, three of whom were dermatologists. The average scores for the accuracy, safety, and agreement of the educational posts were 2.64, 2.83, and 2.55, again all below the midpoint of the scale. Of the four physician-made posts, the average score was 4.25 in all three categories, and of the three dermatologist-made posts, the average scores were 4.83, 5, and 4.83 (Figure 1).

On Instagram and TikTok, TSW is primarily discussed online through personal narratives, encompassing 82% (N = 82) and 84% (N = 84), respectively, of the top 100 TSW posts, while 14% (N = 14) and 15% (N = 15) were categorized as educational. Posts regarding personal experiences ranged in topic from life updates to motivational. However, the most common concern raised was dismissal in the medical field toward TSW leading to patients growing distrustful of physicians. Non-physician-generated educational content frequently promoted non-conventional treatments, such as cold atmospheric plasma (CAP) therapy, no moisture therapy (NMT), and red light therapy, as well as advocated for abstaining from any steroid use (Table 1).

**Table 1:** Frequency of non-conventional treatment mentions in Instagram and TikTok educational posts (N = 29)

Non-conventional treatments	Number of educational posts containing mention, n (%)
Cold atmospheric plasma	5 (17%)
No moisture therapy	5 (17%)
Red light therapy	2 (7%)
High protein diet	2 (7%)
Cryotherapy	1 (3%)
Saltwater exposure	1 (3%)
Tanning bed	1 (3%)

**Figure 2:**
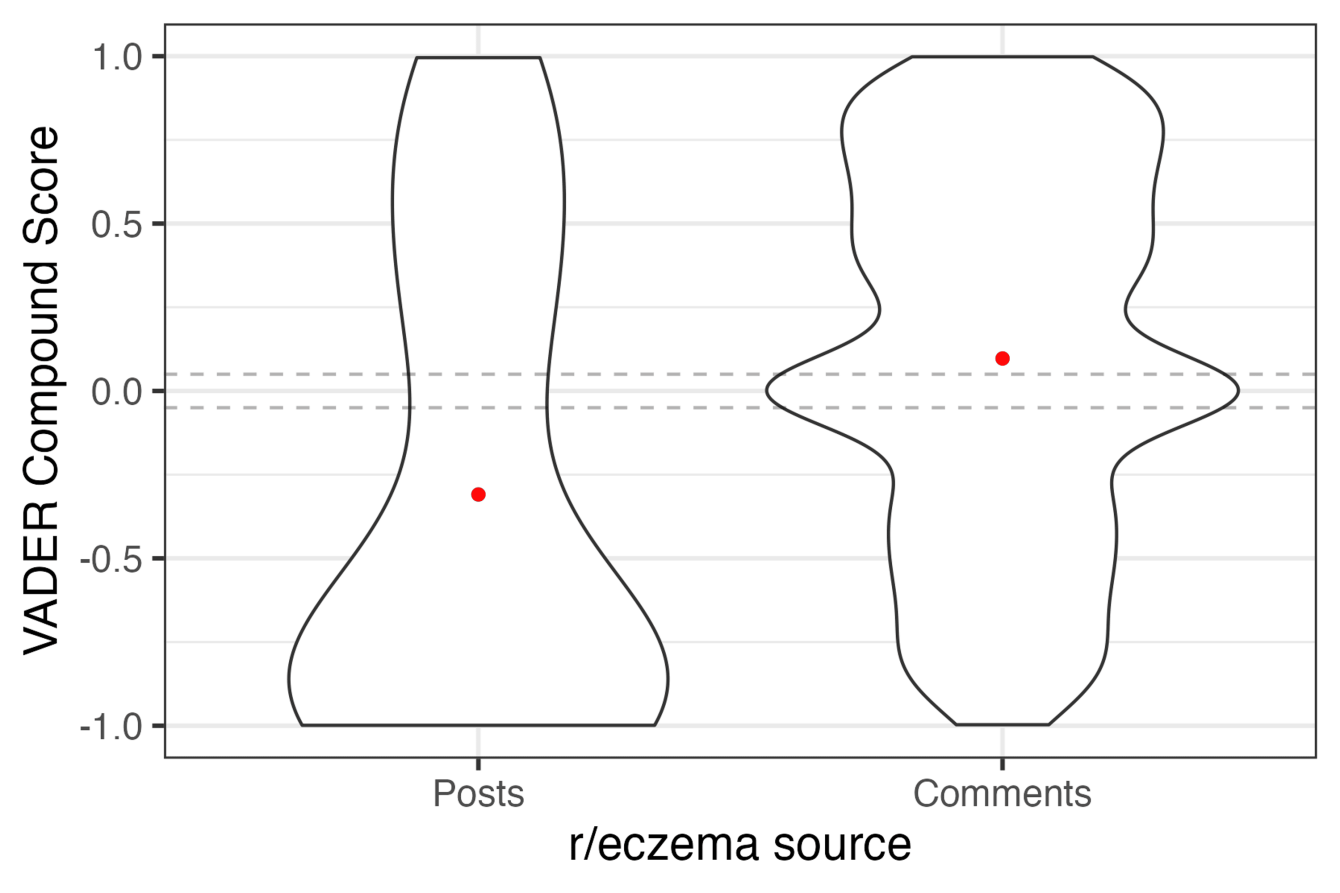
Sentiment analysis of TSW-related posts and comments on r/eczema Violin plots display the distribution of VADER compound sentiment scores for TSW-related posts and comments extracted from the Subreddit r/eczema. The mean is represented by a red point in each group, and the cutoffs for positive and negative sentiment (0.05 and -0.05) are indicated by horizontal dotted lines. TSW: topical steroid withdrawal; VADER: Valence Aware Dictionary and sEntiment Reasoner

In the r/eczema Subreddit, 34 of the top 1,000 posts contained the keywords “TSW” or “topical steroid withdrawal.” The 34 posts had a combined count of 1,757 comments. Obtained from sentiment analysis, the mean VADER compound sentiment score was -0.31 for the posts and 0.10 for the comments (Figure 2).

Twenty-one (62%) of the 34 posts had a negative sentiment score. These posts underwent manual review, and among the 21 posts, 15 (71%) users shared personal experiences, two (10%) users shared how the fear of TSW made them afraid to use TCS, and two (10%) users shared their opinions that TSW was overly diagnosed online and created “fear mongering”.

## Discussion

Much of the discourse surrounding TSW occurs online, primarily through the lens of individuals who suffer from the condition and want to share their personal experiences. While social media may aid patients in finding community and support, the vast majority of TSW-related content on social media platforms is created by non-physicians and/or those without educational and professional training in dermatology. Our analysis found that educational content generated by non-physicians was scored by dermatology residents to be on average below the midpoint in accuracy, safety, and agreement.

Discussions on Reddit related to TSW reveal negative sentiments overall, with a majority of posts reflecting shared experiences and frustrations associated with feeling unheard by medical professionals. Other perspectives shared included hesitancy toward the usage of TCS due to the fear of TSW and its associated challenges, as well as discussions regarding the role that “fear-mongering” and self-diagnoses may play in TSW.

The promotion of information that is inaccurate about TWS may lead to patients pursuing treatments that are ineffective or even harmful. Table 1 describes the non-conventional treatments promoted by online posts, with CAP therapy being the most prevalent.

CAP therapy uses ionized gas at room temperature to deliver reactive species, such as reactive oxygen and nitrogen species, ultraviolet (UV) photons, and electromagnetic emissions, to the skin [14]. Notably, CAP therapy has been described in case reports and small cohorts of patients as an effective treatment for various skin diseases, including wound healing and actinic keratosis [14-17]. Pursuit of this therapy leads patients to relocate to Thailand for several months to undergo consistent treatment using specialized haircare and skincare products formulated and produced by the CAP therapy clinic. These products are unregulated, making it difficult to assess their safety and efficacy, and this process would be costly for many patients.

NMT, referenced on social media as a treatment for TSW, appears to be based on a regimen developed by dermatologist Dr. Kenji Sato, MD, of the Japanese Dermatological Association. His approach, designed for patients with atopic dermatitis, involves withdrawal from both TCS and moisturizers. Dr. Sato has previously investigated TCS-free approaches in patients with atopic dermatitis [18]. However, NMT has not been rigorously tested and was intended specifically for atopic dermatitis, not TSW. Online claims that moisturizing worsens TSW and that water intake should be limited are concerning. Demonstrated in clinical studies to significantly improve the skin barrier, twice-daily moisturization is recommended by international guidelines for the management of atopic dermatitis [19]. The benefits of moisturization have also been shown in the prevention and long-term management of atopic dermatitis. It is also significant to note that dehydration can have deleterious effects on kidney function and cardiovascular health [20,21].

Other less-cited treatments promoted on social media included red light therapy, cryotherapy, high protein intake, saltwater exposure, and tanning bed use. Red light therapy, or low-level laser therapy, stimulates biological activity through light absorption [22]. It has shown benefits in acne treatment, wound healing, and pain management, but there is currently no evidence to support its use in TSW [22,23]. Cryotherapy is the use of freezing temperatures to freeze and destroy abnormal tissue [24]. It is utilized to treat a variety of skin lesions, including basal and squamous cell carcinomas that have low-risk features, but lacks evidence of TSW management [24]. In regard to protein intake, one study reported a negative correlation between plant-based protein intake and atopic dermatitis risk in Chinese adults from Singapore and Malaysia [25]. Further research is needed to evaluate the role of a high protein diet in TWS management. Saltwater application has shown anti-inflammatory effects in mice, yet its therapeutic relevance in TSW remains untested [26,27]. Meanwhile, tanning bed use is not recommended due to its established risks for UV-induced skin damage and skin cancer risk [28].

Dermatologist-generated content on TSW primarily recommended dupilumab, a monoclonal antibody that blocks interleukin 4 and 13 signaling. Dupilumab has been shown to effectively treat atopic dermatitis among other skin conditions and, in case studies, has shown promising results for TSW [6,29,30]. Further studies are necessary to support its use. Additional non-steroidal treatments used for TSW management in the literature include low-dose oral antibiotics, antihistamines, topical calcineurin inhibitors, topical agents for neuropathic pain (amitriptyline, gabapentin), topical emollients and moisturizers, and systemic immunosuppressants. In a cohort of 19 patients with TSW, all patients following conventional management noted significant improvements in their skin [6]. It was noted that patients managed with dupilumab showed clearance of their TSW and atopic dermatitis. The two patients managed with systemic immunosuppressants showed similar findings.

While patient experiences are valuable and can inspire research, it is crucial to ensure that treatment approaches are grounded in scientific evidence to protect patient safety. High-quality studies are needed to better understand if currently unsubstantiated treatments have comparable, or improved, efficacy to current evidence-based treatments. This study is limited by the descriptive nature of social media data, as posts often lack detailed clinical information. Additionally, our analysis is restricted to specific keywords on Instagram, TikTok, and Reddit that may not capture all TSW-related content. Despite these limitations, a key strength of this study is that it provides insight into real-world patient experiences and perspectives on TSW, highlighting trends and information shared online that are not routinely documented in literature.

## Conclusions

This study explores the sentiments and claims regarding TWS on social media and their potential impact on patients with dermatologic conditions. Our findings demonstrate that non-physician-generated content on TSW was typically rated low in accuracy, safety, and agreement by dermatologists. Despite limited evidence, patients frequently pursue alternative therapies. Since TSW is not officially recognized in the ICD-10, many dermatologists may not classify the constellation of symptoms following TCS cessation as a distinct disease, leaving patients feeling dismissed.

To address this, dermatologists can leverage the lack of physician-generated content on TSW to provide accurate, safe, and accessible information. Those with pre-established social media platforms are particularly well-positioned to lead this discourse. Moreover, establishing clear diagnostic criteria and encouraging evidence-based treatments, such as dupilumab, would better equip physicians and patients to address TSW in a safe and constructive manner. As online discourse continues to shape patient decisions, it is essential for the medical community to prioritize research into TSW and its treatment to ensure safe and effective treatment options for those affected.
